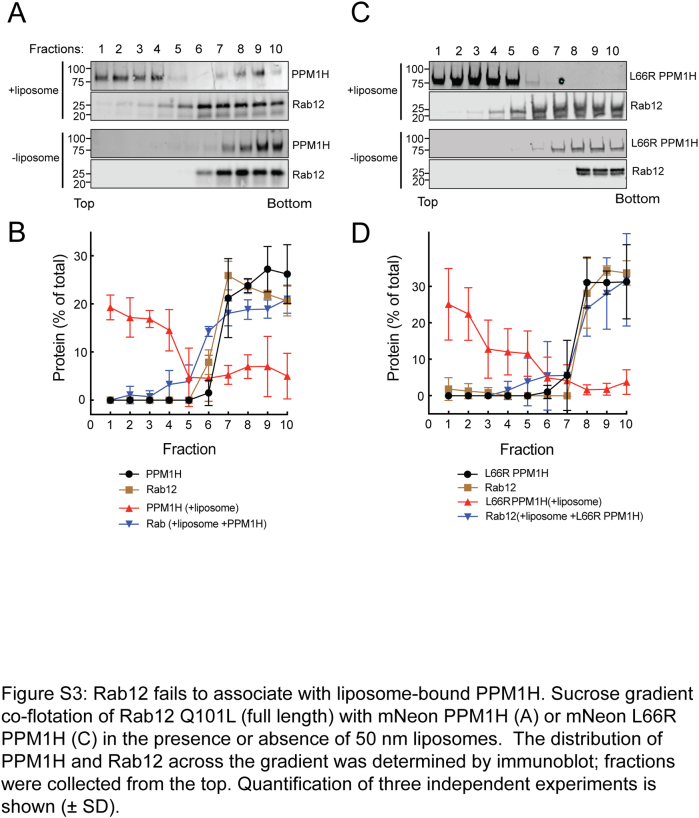# Allosteric regulation of the Golgi-localized PPM1H phosphatase by Rab GTPases modulates LRRK2 substrate dephosphorylation in Parkinson’s disease

**DOI:** 10.1016/j.jbc.2025.110679

**Published:** 2025-09-03

**Authors:** Ayan Adhikari, Aashutosh Tripathi, Claire Y. Chiang, Pemba Sherpa, Suzanne R. Pfeffer

**Affiliations:** 1 Department of Biochemistry, Stanford University School of Medicine, Stanford, California, United States; 2 Aligning Science Across Parkinson’s (ASAP) Collaborative Research Network, San Francisco, California, United States

**Keywords:** phosphatase, leucine-rich repeat kinase 2, enzyme inhibitor, Parkinson's disease, Rab GTPase

## Abstract

PPM1H phosphatase reverses Parkinson’s disease–associated, leucine-rich repeat kinase 2 (LRRK2)–mediated, Rab GTPase phosphorylation. We showed previously that PPM1H relies on an N-terminal amphipathic helix for Golgi membrane localization, and this helix enables PPM1H to associate with liposomes *in vitro*; binding to highly curved liposomes activates the phosphatase activity of PPM1H. We show here that PPM1H also contains an allosteric binding site for its nonphosphorylated reaction products, Rab8A and Rab10. Microscale thermophoresis revealed that PPM1H binds thiophosphorylated Rab8A at the active site with a *K*_*D*_ of ∼1 μM; binding of Rab8A and Rab10 to an alternative site is of similar affinity and is not detected for another LRRK2 substrate, Rab12. Nonphosphorylated Rab8A or Rab10 inhibit PPM1H phosphatase reactions at concentrations consistent with their measured binding affinities and fail to inhibit PPM1H L66R phosphatase reactions. Independent confirmation of nonphosphorylated Rab binding to PPM1H was obtained by sucrose gradient coflotation of nonphosphorylated Rabs with liposome-bound PPM1H. Finally, Rab8A or Rab10 binding also requires PPM1H’s amphipathic helix, without which the interaction affinity is decreased about sixfold. These experiments indicate that Golgi-associated Rab proteins contribute to the localization of PPM1H, and nonphosphorylated Rabs regulate PPM1H phosphatase activity *via* an allosteric site. Targeting this site could represent a strategy to enhance PPM1H-mediated dephosphorylation of LRRK2 substrates, offering a potential therapeutic approach to counteract LRRK2-driven Parkinson’s disease.

Activating mutations in leucine-rich repeat kinase 2 (LRRK2) are a major cause of inherited Parkinson’s disease (1, 2). LRRK2 phosphorylates a subset of the 65 human Rab GTPases at a critical threonine or serine residue within the Rab’s so-called switch 2 motifs (3, 4). Rab8A, Rab10, and Rab12 are broadly expressed and prominent LRRK2 substrates, whereas Rabs 3, 29, 35, and 43 show more tissue-specific expression and appear to be phosphorylated at lower stoichiometries (5). It is important to note that at steady state, only a few percent of even the most abundant Rab substrates are phosphorylated (5), and the phosphate modification shows a high rate of turnover, in some cases, with a half-life of as little as 1 to 2 min (6, 7).

Rab phosphorylation blocks the ability of Rabs to bind their normal effector, partner proteins, to be activated by cognate guanine nucleotide exchange factors, and to be retrieved from membranes by GDP dissociation inhibitor proteins (3, 4). Thus, phosphorylation inhibits normal Rab function. However, once phosphorylated, Rabs gain the capacity to bind to phosphoRab (pRab)-specific effectors, with dominant effects leading to cellular pathology. pRab effectors identified to date include RILPL1 (Rab interacting lysosomal protein-like 1), RILPL2 (Rab interacting lysosomal protein-like 2), JIP3 (c-Jun N-terminal kinase–interacting protein 3), JIP4 (c-Jun N-terminal kinase–interacting protein 4), and MyoVa (Myosin Va) proteins (3, 8, 9).

Berndsen *et al.* (6) discovered PPM1H as a Rab-specific phosphatase that can reverse LRRK2-mediated Rab phosphorylation. Multiple lines of evidence confirm the role of PPM1H in pRab biology. Loss of PPM1H phenocopies hyperactivation of LRRK2 in cell culture and mouse brain. Upon addition of LRRK2 inhibitors, cells lacking PPM1H display a threefold higher basal level of Rab8 and Rab10 phosphorylation than control cells. Finally, unbiased mass spectrometry of proteins bound to a substrate-trapping PPM1H mutant showed strong enrichment for Rab10, Rab8A, and Rab35 (6).

PPM1H relies upon an N-terminal, amphipathic helix for its membrane association in cells (10). In addition, using purified proteins, we showed that the amphipathic helix can mediate liposome association and confers the capacity of PPM1H to be activated by highly curved membranes (10). In cells, PPM1H is localized near its pRab8A and pRab10 substrates (6, 10) and it can act on pRab12 if the two proteins are artificially colocalized on mitochondrial surfaces (10). These experiments highlight the importance of enzyme and substrate colocalization contributing to apparent substrate specificity in cellular assays.

In our initial, *in vitro* PPM1H-catalyzed Rab dephosphorylation assays, we noticed that the reactions did not go to completion. Troubleshooting revealed that our pRab10 substrate preparations were not 100% phosphorylated, and this led to our discovery that PPM1H is inhibited by nonphosphorylated reaction products. AlphaFold 3 (11) modeling helped us identify a potential allosteric Rab GTPase binding site located behind the PPM1H active site. We present here our biochemical characterization of allosteric inhibition of PPM1H phosphatase.

## Results and discussion

Figure 1 shows an AlphaFold 3 model (11, 12) for PPM1H bound to pRab10 in the presence of nonphosphorylated Rab10 protein (pTM = 0.57). The structure of PPM1H was first reported by Waschbüsch *et al.* (13); AlphaFold correctly orients the phosphorylated Rab10 such that the substrate phosphate points directly into the enzyme active site. (Note that PPM1H is a stable dimer (13); we show the interactions for one half of the dimeric model for clarity. None of these interactions would be obscured in a dimer model.) Significant surface interaction is also seen between the FLAP domain and the pRab substrate, consistent with the role of the FLAP in substrate selectivity (12). In this representation, we have deleted loops #1 and #2 (residues 115–133 and 204–217) that do not influence protein localization or enzyme activity (10) and were not resolved in the crystal structure ((13); Fig. S1*A*). The PPM1H L66 side chain is predicted to contribute to nonphosphorylated Rab10 binding. Note the location of the PPM1H N-terminal, amphipathic alpha helix that will be discussed later. Also indicated is R338 in the FLAP domain that contributes to substrate binding (13).

**Figure 1:**
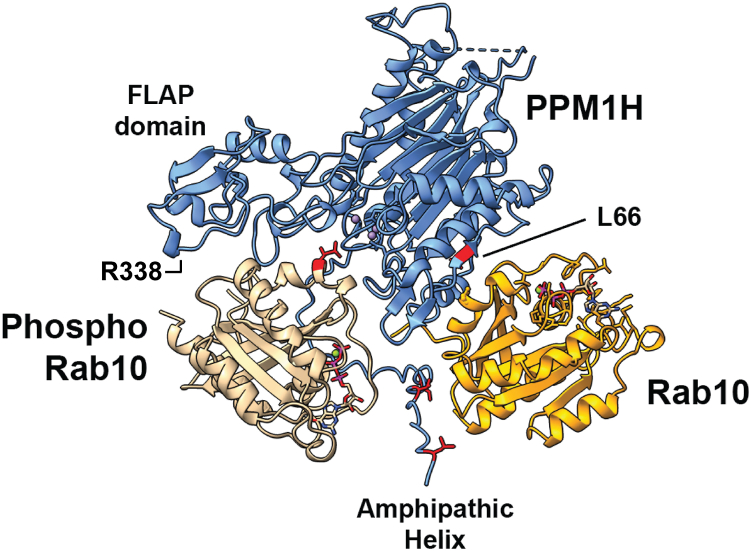
**AlphaFold 3** (11) **model** (12) **of PPM1H bound to its pRab10 substrate and non-pRab10 allosteric regulator (loops #1 and #2 were removed for simplicity; see**Fig. S1***A* for comparison).** The PPM1H FLAP domain, amphipathic helix, and Leu66 and R388 residues are indicated. PPM1H (*blue*); pRab10 (*beige*); and Rab10 (*goldenrod*). pRab, phosphoRab.

To test the validity of the AlphaFold model, we engineered a fluorescent version of full-length PPM1H, in which we inserted mNeon into loop#1 at position 125. AlphaFold 3 modeling of PPM1H with and without this insertion (Fig. S1) indicated that the addition of mNeon would not alter overall PPM1H protein structure. HisSUMO-mNeon PPM1H, HisSUMO-mNeon PPM1H L66R or R338A, and HisSUMO-mNeon Δ37 PPM1H were expressed in bacteria and purified by cobalt affinity and size-exclusion chromatography for further analysis; the HisSUMO portions were removed by Ulp1 SUMO protease cleavage prior to gel filtration (Fig. S1, *B*–*E*). As reported previously (13), PPM1H behaved as a dimer upon gel filtration, thus this likely represents the physiologically relevant state.

Figure 2 compares the binding of PPM1H to Rab10 (Fig. 2*A*), Rab8A (Fig. 2*B*), and Rab12 (Fig. 2*C*) monitored using microscale thermophoresis (MST). Nonphosphorylated Rab10 and Rab8A bound PPM1H with a *K*_*D*_ of 1.3 and 1.4 μM, respectively, whereas Rab12 bound much more weakly with a *K*_*D*_ of 7.4 μM (Table 1). In our previous work, Rab effectors that showed comparable low affinities have not colocalized in cells (*cf.* (9)), likely because their concentrations are close to the *K*_*D*_s for WT effector binding. When the same Rabs were tested for binding to PPM1H L66R, the *K*_*D*_ values increased about threefold to 4.4 μM and 5.2 μM for Rabs 10 and 8A (Fig. 2, *D* and *E* and Table 1); binding of Rab12 weakened further to 9.6 μM (Fig. 2*F* and Table 1). Thus, the L66R mutation interferes with non-pRab binding to PPM1H.

**Figure 2:**
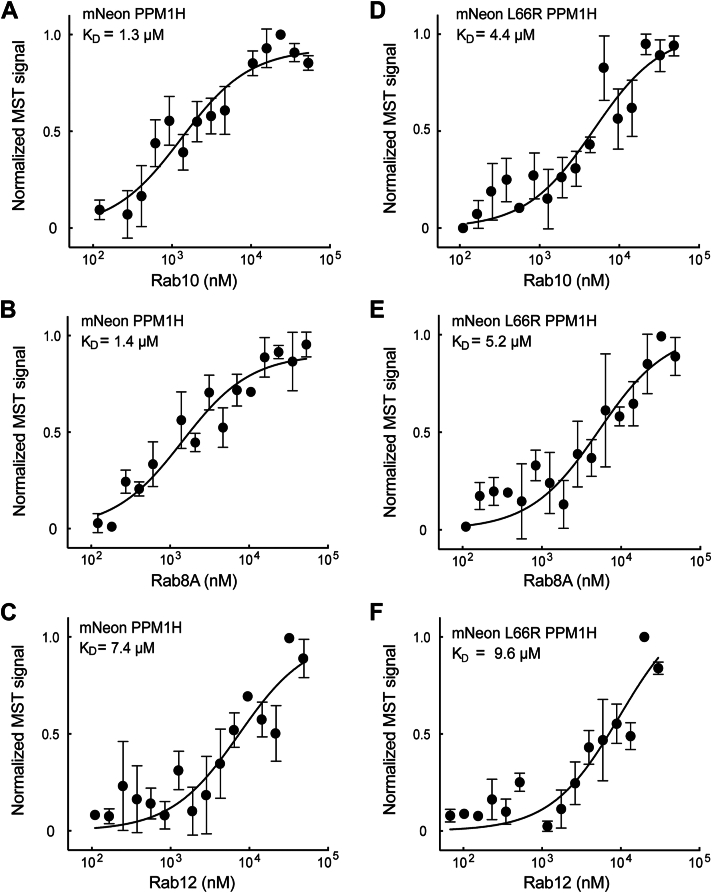
**PPM1H binds nonphosphorylated Rab8A and Rab10 but not Rab12 *via* Leu66.** Microscale thermophoresis of mNeon PPM1H and mNeon L66R PPM1H with Rab10 Q68L (*A* and *D*), Rab8A Q67L (*B*, *E*), or Rab12 Q101L (*C* and *F*). Purified Rab proteins were serially diluted, and mNeon PPM1H or mNeon L66R PPM1H was added (final concentration of 100 nM). Graphs show the mean ± SD from three independent measurements, each using different protein preparations. Values are summarized in Table 1.

**Table 1:** Summary of binding affinities (*K*_*D*_ ± SD)

Rab protein	mNeon PPM1H (μM)	mNeon L66R PPM1H (μM)	mNeon Δ37 PPM1H (μM)	mNeon R338A PPM1H (μM)
Rab8ab8A (Q67L)	1.4 ± 0.98	5.2 ± 3	10.5 ± 6	1.2 ± 0.8
Rab10 (Q68L)	1.3 ± 0.9	4.4 ± 2.7	6.5 ± 4.5	
Rab12 (Q101L)	7.4 ± 3.8	9.6 ± 5.1		
Thio-pRab8A (Q67L)	1 ± 0.6	1.1 ± 0.76		8 ± 4.9
Rab8A WT (GTP bound)	1 ± 0.7			
Rab8A WT (GDP bound)	3 ± 2.1			

To ensure that the non-pRab binding site is distinct from that used by pRab substrates, we used a poorly hydrolyzable, ATPγS thiophosphorylated Rab substrate to enable estimation of the affinity of PPM1H for its phospho-Rab substrate. PhosTag gels showed that the protein was fully modified for use in this experiment and resisted PPM1H action in a 15 min *in vitro* reaction (Fig. S2, *A* and *B*).

**Figure 3:**
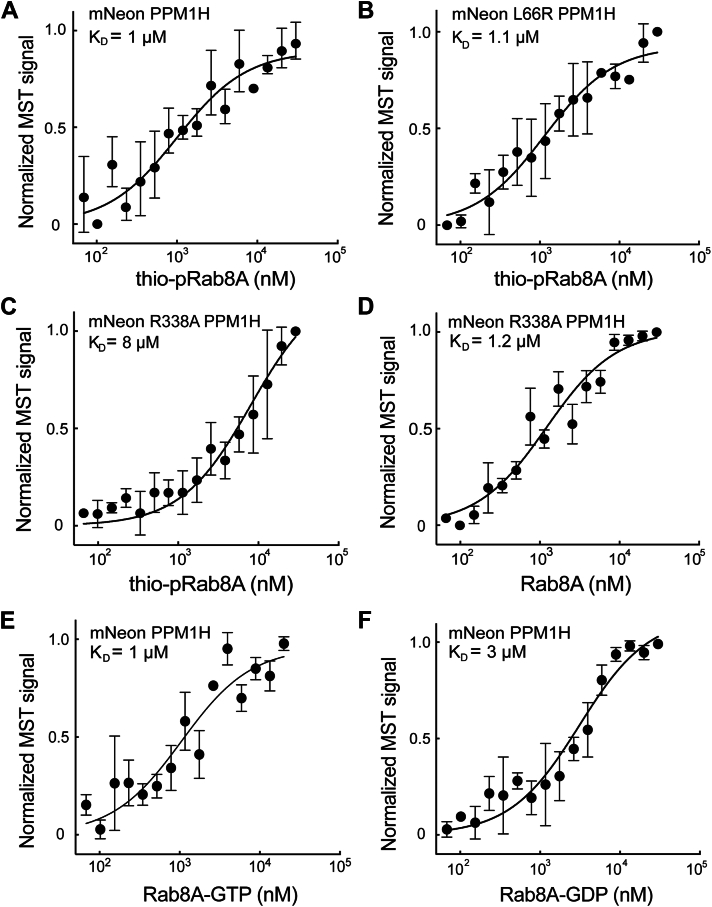
**Binding properties of Rab8A and thiophosphorylated Rab8A to PPM1H.***A* and *B*, thiophosphorylated Rab8A Q67L does not rely on L66 for PPM1H binding. *C* and *D*, thio-phosphorylated Q67L Rab8A but not nonphosphorylated Rab8A binds much less tightly to a PPM1H FLAP domain mutated PPM1H R338A. *E* and *F*, GTP-bound WT Rab8A binds more strongly than GDP-bound WT Rab8A to PPM1H. Various Rab8A or pRab8A proteins were serially diluted and incubated with 100 nM mNeon PPM1H variants as in Figure 2. Graphs represent mean ± SD from three independent experiments using separate protein preparations. pRab, phosphoRab.

Thiophosphorylated Rab8A bound PPM1H with an affinity of 1 μM (Fig. 3*A*); importantly, the *K*_*D*_ was not altered when tested with PPM1H L66R protein *(K*_*D* =_ 1.1 μM; Fig. 3*B*). The FLAP domain residue R338 (Fig. 1) is essential for pRab–substrate binding (13). As expected, PPM1H R338A bound poorly to thiophosphorylated Rab8A (*K*_*D*_ = 8 μM, Fig. 3*C*) but displayed WT binding to unphosphorylated Rab8A (*K*_*D*_ = 1.2 μM, Fig. 3*D*). Thus, unphosphorylated Rab8A binds PPM1H at a distinct, noncatalytic site that involves L66 but not FLAP domain residue R338, consistent with the AlphaFold model (Fig. 1).

The experiments described previously have utilized Rab proteins bearing a Q67/68L mutation that is predicted to represent the GTP-bound state, purified in the continued presence of Mg^++^–GTP. Nevertheless, we also compared the above reported values to the behavior of WT Rab8A protein. In these experiments, Rab8A was loaded with either Mg^++^–GTP or Mg^++^–GDP using EDTA to remove bound nucleotide. Figure 3, *E* and *F* shows that WT GTP-bound Rab8A bound PPM1H approximately threefold more tightly (*K*_*D*_ = 1 μM) than WT GDP–bound Rab8A protein (*K*_*D*_ = 3 μM). Thus, PPM1H displays typical Rab effector behavior with preference for GTP-bound Rab8A. Moreover, the data suggest that our binding determinations carried out with the Q67L Rab proteins are slight underestimates of affinities (1.4 μM for the Q mutant *versus* 1 μM for the WT Rab8A protein).

**Figure 4:**
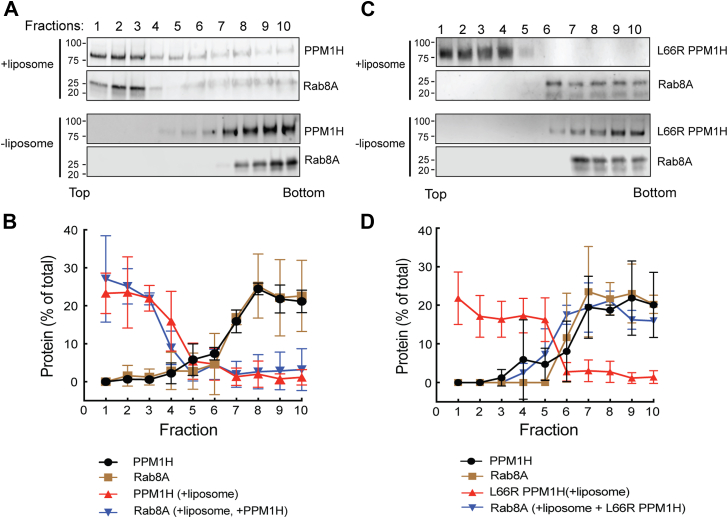
**Rab8A associates with liposome-bound PPM1H.** Sucrose gradient coflotation of His-Rab8A Q67L (full length, nonprenylated) with mNeon PPM1H (*A*, *B*) or mNeon L66R PPM1H (*C*, *D*) in the presence and absence of 50 nm liposomes. The distribution of PPM1H and Rab8A across the gradient was determined by immunoblot; fractions were collected from the *top*. Quantification of three independent experiments is shown (±SD).

These experiments confirm that PPM1H binds nonphosphorylated Rabs *via* at least one residue (L66) that is not needed for pRab substrate binding, consistent with the AlphaFold model (Fig. 1). In a previous work, we showed that full-length PPM1H protein floats to the top of a sucrose gradient in the presence of liposomes prepared using a Golgi-type lipid composition (10). As an independent test of protein–protein interaction, we utilized this method to ask whether liposome-bound PPM1H would be sufficient to cause a nonprenylated, nonphosphorylated Rab binding partner to also float to the top of a sucrose gradient. Figure 4 shows examples of this type of experiment. mNeon PPM1H remained at the bottom of the gradient unless it was allowed to bind to liposomes; similarly, nonprenylated Rab8A was detected at the bottom of the tube unless both PPM1H and liposomes were added (Fig. 4, *A* and *B*). Importantly, Rab8A failed to float with liposomes bearing PPM1H L66R (Fig. 4, *C* and *D*), despite the ability of that PPM1H mutant to bind and float with liposomes. Control experiments showed that nonprenylated Rab8A alone does not float in the presence of liposomes (Fig. S2, *C* and *D*), confirming that its membrane association requires both PPM1H and the L66 residue. In summary, these experiments confirm that nonphosphorylated Rab8A interacts with PPM1H in a manner that requires PPM1H L66.

**Figure 5:**
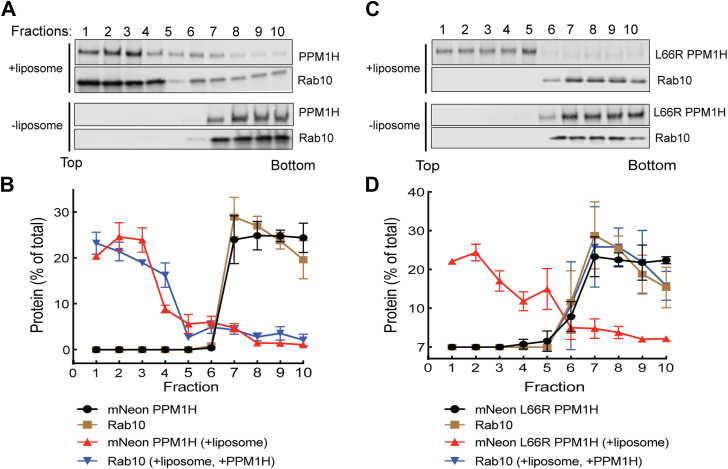
**Rab10 associates with liposome-bound PPM1H.** Sucrose gradient coflotation of Rab10 Q68L (full length) with mNeon PPM1H (*A*) or mNeon L66R PPM1H (*C*) in the presence and absence of 50 nm liposomes. The distribution of PPM1H and Rab10 across the gradient was determined by immunoblot; fractions were collected from the *top*. Quantification of three independent experiments is shown (±SD).

Identical results were obtained in experiments analyzing the ability of PPM1H to bind to nonphosphorylated Rab10 (Fig. 5, *A*–*D*). In contrast, upon sucrose gradient sedimentation, nonphosphorylated Rab12 exhibited only weak binding to liposome-bound WT or L66 mutant PPM1H (Fig. S3, *A*–*D*), consistent with the low affinities determined by MST (Fig. 2, *C* and *F*). Thus, Rab8A and Rab10, but not Rab12, can bind to PPM1H in a manner that requires PPM1H L66.

## Nonphosphorylated Rabs require PPM1H N-terminal sequences for binding

**Figure 6:**
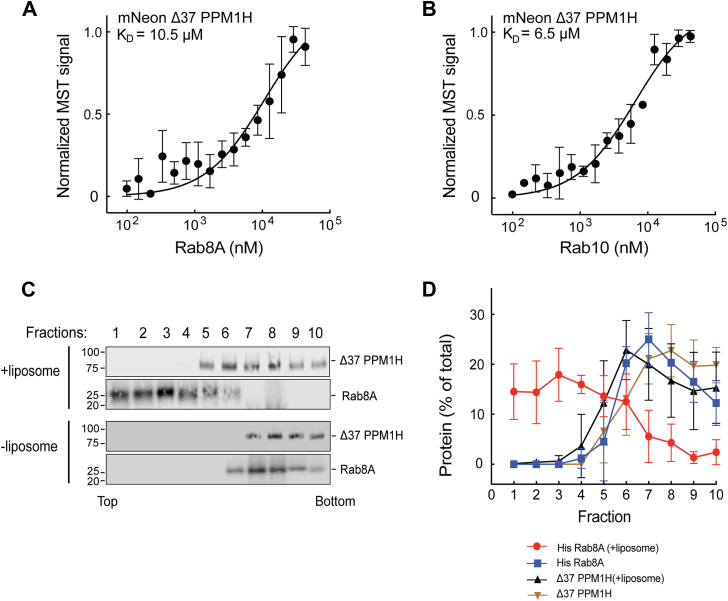
**PPM1H’s N-terminal residues contribute to Rab8A and Rab10 binding.** Microscale thermophoresis of mNeon Δ37 PPM1H with His Rab8A Q67L (*A*) or Rab10 Q68L (*B*). *C*, sucrose gradient coflotation of His Rab8A Q67L with mNeon Δ37 PPM1H in the presence and absence of 50 nm diameter NTA(Ni) containing liposomes. *D*, quantification of three independent experiments is shown (±SD). Ni, nickel; NTA, nitrilotriacetic acid.

We showed previously that deletion of the N-terminal 37 residues caused PPM1H to display an entirely cytosolic localization (10). Thus, at first, it was hard to explain why our newly discovered, relatively high-affinity Rab binding site was not sufficient to localize PPM1H to Rab8A and Rab10-positive Golgi membranes in cells. One possible explanation was that the PPM1H N terminus also contributes to Rab8A and Rab10 binding. To test this, we compared the binding of nonphosphorylated Rab proteins to WT and Δ37-PPM1H. To our surprise, binding of Rab8A and Rab10 to Δ37-PPM1H missing the amphipathic helix decreased from 1.3 and 1.4 μM, respectively, to 10.5 and 6.5 μM (Fig. 6, *A* and *B* and Table 1). Thus, in addition to L66, PPM1H’s N-terminal 37 residues are also needed for both liposome association and non-pRab protein binding.

As an independent measure of the importance of the PPM1H N terminus in Rab binding, we artificially anchored Rab8A-6XHis onto the surface of nitrilotriacetic acid (NTA)–lipid–containing liposomes and tested whether binding at the PPM1H allosteric site would be sufficient to float Δ37 PPM1H. As shown in Figure 6, *C* and *D*, despite efficient flotation of Rab8A-6XHis with the NTA–lipid-containing liposomes, Δ37 PPM1H failed to float to the top of the sucrose gradient, consistent with its weak affinity for Rab8A determined by MST. Altogether, these experiments confirm the importance of the PPM1H amphipathic N- terminus, both for binding to highly curved membranes and to nonphosphorylated Rab proteins. It is not alone sufficient for binding because L66R PPM1H also failed to bind Rab8A and Rab10 proteins, despite the presence of the PPM1H N-terminus. Because PPM1H is a dimer, these results might be explained if one N-terminal sequence engages the liposome, whereas the other engages the Rab GTPase; alternatively, it may be possible that one face of the amphipathic helix can be anchored in the liposome membrane, whereas its exposed surface interacts with Rab proteins. It is worth noting that among the PPM phosphatase family, PPM1J, but not PPM1M, also possesses an N-terminal amphipathic helix.

## Allosteric inhibition of PPM1H

**Figure 7:**
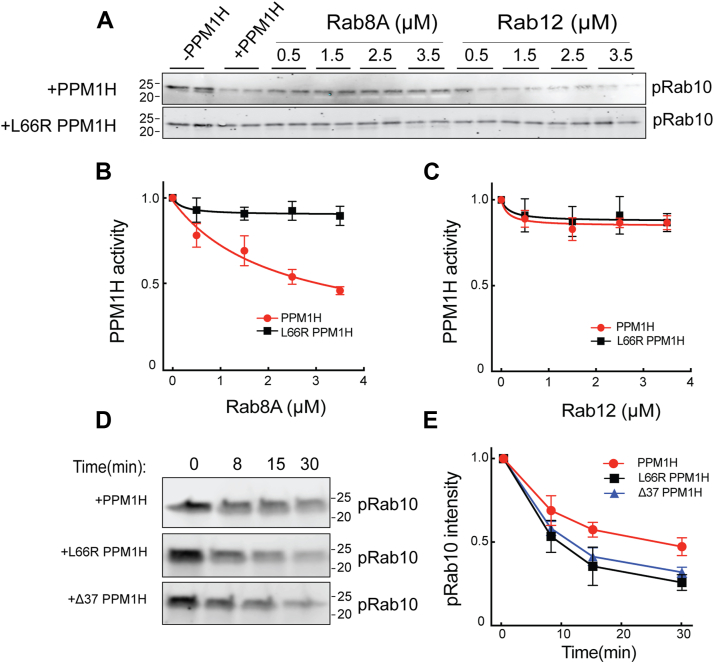
**Rab8A, but not Rab12, inhibits PPM1H activity.***A*, anti-pRab10 immunoblot analysis of dephosphorylation by PPM1H (*upper gel*) or L66R PPM1H (*lower gel*). *B* and *C*, data were plotted as PPM1H activity, with maximum activity equal to the amount of pRab10 dephosphorylation seen in the absence of non-pRab inhibitor (lanes 3 and 4 of each gel) compared with reactions lacking PPM1H (lanes 1 and 2). *D*, rate of pRab10 phosphatase activity (monitored at *t* = 0, 8, 15, and 30 min) for mNeon-PPM1H, mNeon-L66R PPM1H, and mNeon Δ37 PPM1H. *E*, quantification of pRab10 intensity from (*D*), with maximum intensity detected at 0 min normalized to a value of 1. Error bars represent SD from three independent experiments; representative gels are shown. *Black line*, mNeon L66R PPM1H activity; *red line*, mNeon PPM1H activity; and *blue line*, mNeon Δ37 PPM1H activity. pRab, phosphoRab.

Rab10 dephosphorylation was monitored *in vitro* using purified proteins in conjunction with immunoblot analysis using a pRab10-specific antibody. As shown in Figure 7*A*, pRab10 was readily detected, and as expected, its abundance decreased upon addition of purified PPM1H enzyme. Figure 7, *B* and *C* show replots of the data to display PPM1H *activity* rather than loss of pRab10 protein: full activity is defined as the amount of pRab remaining in the presence of 100 nM PPM1H protein (lanes 3 and 4, Fig. 7*A*). When reactions also contained micromolar amounts of unphosphorylated Rab8A protein, a concentration-dependent inhibition of phosphatase activity was observed (Fig. 7, *A* and *B*). In contrast, addition of comparable amounts of Rab12 failed to inhibit PPM1H enzyme (Fig. 7*C*), consistent with the very weak interaction of Rab12 with PPM1H (Figure 2, FigS3 and Fig. S3). The concentrations of Rab8A or Rab10 needed to achieve 50% inhibition were entirely consistent with *K*_*D*_ determinations (Fig. 2 and Table 1). Thus, PPM1H displays allosteric inhibition by its cellular Rab8A and Rab10 substrates. Note that Rab12 is not an endogenous, cellular substrate for PPM1H enzyme (6, 7).

## L66R and Δ37 PPM1H are more catalytically active than WT PPM1H

If non-pRabs inhibit PPM1H, one would predict that PPM1H mutants that cannot bind non-pRabs should display higher catalytic activity. Figure 7, *D* and *E* shows that this is indeed the case: both the L66R and Δ37 PPM1H variants exhibited increased phosphatase activity relative to WT PPM1H, as indicated by a ∼2-fold more rapid reduction in pRab10 over time.

In summary, we have reported here the existence of an allosteric binding site for nonphosphorylated, substrate Rab GTPases on PPM1H enzyme and shown that binding at this site inhibits PPM1H catalytic activity. In addition to regulating PPM1H activity, Rab binding appears to contribute to membrane localization of PPM1H *via* the phosphatase’s N-terminal amphipathic helix, since Rab binding and localization both rely on this sequence. Does this mean that membrane-associated PPM1H is held in an inactive conformation?

**Figure 8:**
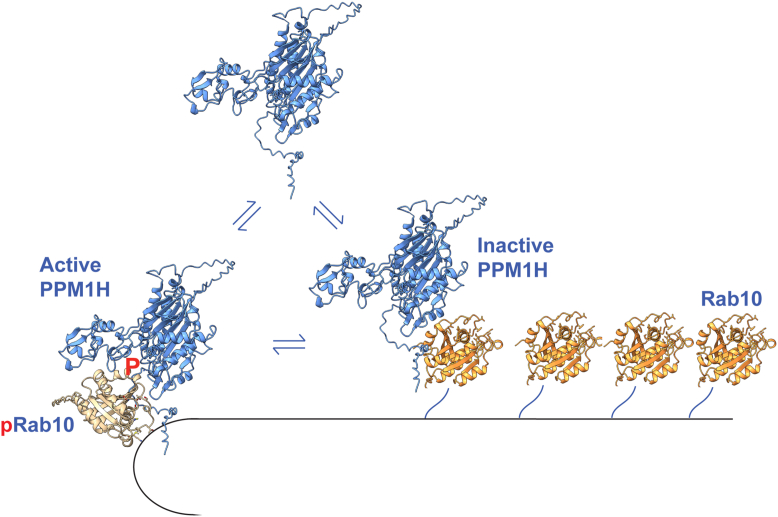
**A model for PPM1H membrane recruitment and allosteric regulation.** For details, see text.

We showed previously that in the absence of nonphosphorylated Rab proteins, PPM1H is activated upon amphipathic helix binding to highly curved (50 nm diameter) liposomes (10). In that study, we concluded that such binding would enrich the enzyme at highly curved Golgi rims. We speculate that an initial Rab protein interaction brings PPM1H to the correct membrane compartment, in this case, the Golgi complex (Fig. 8). PPM1H shows similar binding affinity for pRabs and non-pRabs *via* independent binding sites described here. Why would an enzyme bind substrate and inhibitor with similar affinities? pRab proteins represent only a few percent of total Rab proteins (5). Considering that cells contain ∼1 million Rab8A or Rab10 proteins and only about 10,000 PPM1H proteins, we propose that the more abundant Rab8A and Rab10 proteins recruit PPM1H to membrane domains where these Rabs are most concentrated; this will enhance the probability of PPM1H encountering rare, phosphorylated Rabs. Although modeling suggests that PPM1H can bind both a pRab and a nonphosphorylated Rab simultaneously (Fig. 1), it seems more likely that the two binding sites operate independently to enable fine tuning of pRab levels in cells. A prediction of this model is that PPM1H will be more active in locations were the corresponding non-pRabs are less abundant and therefore, less likely to inhibit phosphatase activity. An example is under conditions of lysosome stress when pRabs are detected on lysosomes where they are not normally found (14, 15, 16). Future experiments will be needed to fully explore this model in greater detail. Altogether, these findings identify a dual role for Golgi-localized, nonphosphorylated Rab GTPases in the spatial localization and regulation of PPM1H, revealing a layer of phosphatase control that could be leveraged to enhance dephosphorylation of LRRK2 substrates.

## Experimental procedures

### Cloning and plasmids

DNA constructs were amplified in *Escherichia coli* DH5α and purified using mini prep columns (EconoSpin). DNA sequencing was performed by MC LAB (https://mclab.com/). pET15b His-MST3 was a generous gift from Amir Khan (Trinity College, Dublin). mNeon was cloned into loop 1 (residues 115–133) of pET15b His SUMO PPM1H and pET15b His SUMO Δ37 PPM1H backbones. The PPM1H L66R or R338A point mutation was introduced using site-directed mutagenesis. His Rab8A Q67L was subcloned from HA-Rab8A (DU35414; MRC PPU) into the pET14b vector. His SUMO Rab10 (full length) Q68L and His SUMO Rab12 (full length) Q101L were cloned from pET15b Rab10 Q68L and pQE 80L Rab12 (Q101L), respectively, into the pET15b backbone. All cloning and subcloning was performed using Gibson assembly (https://www.protocols.io/view/gibson-assembly-cloning-eq2lyjwyqlx9/v1).

### Protein purification

The following protocol describes purification of His SUMO mNeon PPM1H, His SUMO mNeon Δ37 PPM1H, His SUMO mNeon L66R or R338A PPM1H, His SUMO Rab10 (Q68L), His Rab8A (Q67L), His MST3, and His SUMO Rab12 (Q101L): https://www.protocols.io/view/ppm1h-purification-from-e-coli-d67v9hn6. All Rab proteins were of full length and nonprenylated. Rab Q mutants were used throughout this study; these favor the GTP-bound state, making it easier to ensure that the Rabs are as active as possible in these experiments. For Rab protein purification, 20 μM GTP was included in all buffers. Briefly, bacterial cultures were grown at 37 °C in Luria broth and induced with 0.3 mM isopropyl-1-thio-β-d-galactopyranoside when the absorbance at 600 nm reached 0.5 to 0.6. Cultures were harvested after overnight growth at 18 °C. The cell pellets were resuspended in ice-cold lysis buffer containing 50 mM Hepes (pH 8.0), 10% glycerol, 500 mM NaCl, 10 mM imidazole, 5 mM MgCl_2_, 0.2 mM Tris(2-carboxyethyl)phosphine hydrochloride (TCEP), 20 μM GTP, and an EDTA-free protease inhibitor cocktail (Roche; #69040300). Bacterial cells were lysed using an Emulsiflex-C5 apparatus (Avestin) at 10,000 psi and centrifuged at 40,000 rpm for 45 min at 4°C using a Beckman Ti45 rotor. The clarified lysate was filtered through a 0.2 μm filter (Nalgene) and passed over a HiTrap TALON crude 1 ml column (Cytiva; #28953766). The column was washed with lysis buffer until the absorbance returned to baseline, and proteins were eluted using a gradient of 100 to 500 mM imidazole in the same buffer. Peak fractions were identified by SDS-PAGE using 4% to 20% precast protein gels. For His-MST3 (mammalian STE20-like protein kinase 3) and His Rab8A, the eluted proteins were concentrated by Centricon filtration before being applied to a Superdex 75 10/300 GL column (Cytiva; #29148721).

TALON purified, His-SUMO-tagged proteins were dialyzed overnight together with Ulp1 SUMO protease (100 ng/mg of protein) to remove the His-SUMO tag. The following day, the dialyzed proteins were collected, concentrated, and applied onto a Superdex 200 10/300 GL column (Cytiva; #28990944) fitted in series with an additional 1 ml HiTrap TALON crude column (Cytiva; #28953766) to remove uncleaved His-SUMO protein, the His-tagged Ulp1 SUMO protease, and the His-SUMO tag. Where noted, nucleotide exchange was performed as described (17). Briefly, 2.2 mg purified WT Rab8A protein was incubated in 20 mM Hepes (pH 7.5), 100 mM NaCl, 5 mM EDTA, and 40 mM GDP or GTP at room temperature for 30 min to facilitate nucleotide release. The reaction was terminated by the addition of 10 mM MgCl_2_ to stabilize rebound nucleotide. Excess nucleotide was removed using a desalting column equilibrated with a buffer containing 50 mM Hepes (pH 8), 150 mM NaCl, 5 mM MgCl_2_, 10% glycerol, and 50 μM GDP or GTP.

### Microscale thermophoresis

Protein–protein interactions were analyzed using MST on a Monolith NT.115 instrument (NanoTemper Technologies) as described in detail: dx.doi.org/10.17504/protocols.io.bvvmn646. His-tagged Rab8A Q67L was incubated overnight at 4 °C with His-tagged MST3 kinase in a reaction buffer containing 50 mM Hepes (pH 8), 100 mM NaCl, 5 mM MgCl_2_, 2 mM ATP or ATPγS, 100 μM GTP, 0.5 mM TCEP, 10% glycerol, and 5 μM bovine serum albumin. In all experiments, the unlabeled (Rab12 Q101L, Rab10 Q68L, His Rab8A Q67L, and His pRab8A Q67L) protein partner was titrated against a fixed concentration of fluorescent mNeon PPM1H, mNeon Δ37 PPM1H, or mNeon L66R PPM1H (100 nM). Sixteen serial dilutions of the unlabeled protein partner were prepared to generate a complete binding isotherm. Binding reactions were carried out in a reaction buffer in 0.5 ml Protein LoBind tubes (Eppendorf) and incubated in the dark for 30 min before being loaded into NT.115 premium–treated capillaries (NanoTemper Technologies).

Measurements were taken using a blue LED set at 30% excitation power (excitation: 450–480 nm; emission: 515–570 nm) and an IR-laser at 60% power for 30 s, followed by 5 s of cooling. Data were analyzed using NTAffinityAnalysis software (NanoTemper Technologies), where raw fluorescence data were used to generate binding isotherms. These isotherms were fitted using both NanoTemper software and GraphPad Prism to calculate the dissociation constant (*K*_*D*_) *via* nonlinear regression. Binding affinities derived from both methods were consistent.

### Liposome preparation

A detailed step-by-step protocol can be found at: https://doi.org/10.17504/protocols.io.5qpvo3ke7v4o/v1. Briefly, liposomes were prepared using a Golgi-like lipid composition consisting of (18:0–20:4)PC (1-stearoyl-2-arachidonoyl-*sn*-glycero-3-phosphocholine), (18:0–20:4)PI (1-stearoyl-2-arachidonoyl-*sn*-glycero-3-phosphoinositol), (18:0–18:2)PS (1-stearoyl-2-linoleoyl-*sn*-glycero-3-phospho-L-serine), (18:1) plus PI(4)P, and cholesterol in a molar ratio of 78:7:5:1:9 (all lipids from Avanti Polar Lipids). For the flotation assay of His-Rab8A (Q67L) with mNeon Δ37 PPM1H, the liposome mixture included (18:1)DGS NTA 1,2-dioleoyl-*sn*-glycero-3-[(*N*-(5-amino-1-carboxypentyl)iminodiacetic acid)succinyl] (nickel salt) (Ni2+) and was prepared using a molar ratio of 76:7:5:1:9:2.

The lipid mixtures, dissolved in chloroform, were dried under a nitrogen stream to form a thin film. This film was then resuspended in 50 mM Hepes (pH 7.5) and 120 mM KCl. The suspension underwent two brief 5-s sonication cycles in a bath sonicator and was subsequently extruded through 0.4 μm, 0.1 μm, and 0.05 μm polycarbonate filters (21 passes each) using a hand extruder (Avanti). The final liposome suspension had a lipid concentration of 15 mM.

### Sucrose density gradient coflotation

A detailed protocol is available: dx.doi.org/10.17504/protocols.io.q26g79o2kvwz/v1. Liposome coflotation assays were conducted by incubating 3 μM Rab10, Rab12, or His Rab8A with 1.5 mM, 50-nm diameter liposomes and 1 μM mNeon PPM1H, mNeon L66R PPM1H, or mNeon Δ37 PPM1H for 30 min at 30 °C in a total volume of 33 μl using HKM buffer (20 mM Hepes, pH 7.5, 150 mM potassium acetate, 1 mM magnesium chloride, and 20 μM GTP). Next, 167 μl of 60% (w/v) sucrose was added and mixed to adjust the solution to 50% sucrose. The sample was then overlaid with 200 μl of 25% (w/v) sucrose and 100 μl of HKM buffer. The samples were centrifuged at 45,000 RPM for 3 h using a Ti55 swinging bucket rotor. Ten fractions (50 μl each) were manually collected from1 the top of the gradient using a micropipette and analyzed by immunoblotting using a rabbit anti-PPM1H antibody. Rab10, Rab12, and Rab8A were detected using mouse anti-Rab10, anti-Rab12, and anti-Rab8A antibodies, respectively.

### *In vitro* PPM1H phosphatase assay

A detailed protocol is available: dx.doi.org/10.17504/protocols.io.5jyl8d4j7g2w/v1. Briefly, untagged Rab10 Q68L (full length) was incubated overnight at 4 °C with His-MST3 kinase in a reaction buffer containing 50 mM Hepes (pH 8), 100 mM NaCl, 5 mM MgCl_2_, 2 mM ATP, 100 μM GTP, 0.5 mM TCEP, 10% glycerol, and 5 μM bovine serum albumin to achieve Rab10 phosphorylation. The following day, His-MST3 kinase was removed by passing the reaction mixture through a 1 ml syringe column containing 100 μl (50%) nickel–NTA slurry, and the flowthrough containing phosphorylated Rab10 was collected. Next, 1.5 μM phosphorylated Rab10 was incubated with 100 nM mNeon PPM1H (or 100 nM mNeon L66R PPM1H), in the presence of 0.5 μM, 1.5 μM, 2.5 μM, and 3.5 μM of unphosphorylated Rab8A and Rab12 with 50 nM liposomes, at 30 °C for 15 min. The reactions were stopped by adding a 5X SDS-PAGE sample buffer. Samples were analyzed by immunoblotting to assess Rab10 dephosphorylation using anti-pRab10 (1:1000 dilution) antibody. Note that MST3 kinase was used to phosphorylate Rab10 in place of LRRK2, which is difficult to purify in large amounts and biochemically labile. In contrast, MST3 kinase can be produced in milligram quantities in a highly active form and generates the same pT73 Rab10 product.

To compare the phosphatase activity of mNeon PPM1H, mNeon L66R PPM1H, and mNeon Δ37 PPM1H, phosphorylated Rab10 (1.5 μM) was incubated with 100 nM of each PPM1H variant at 30 °C for 0, 8, 15, and 30 min in a total volume of 15 μl. Reactions were terminated by addition of 5X SDS-PAGE sample buffer. Samples were analyzed by immunoblotting using anti-pRab10 antibody (1:1000 dilution) to assess the extent of dephosphorylation. The anti-pRab10 antibody (Abcam; #230261) has been extensively validated and is widely utilized in the field for detecting phosphorylated Rab10 (18). LICOR scanning and the Image J2 gel analysis (19) feature were used to quantify fluorescent second antibody levels on immunoblots.

### Data analysis

Data analysis was carried out using GraphPad Prism, version 9 for Mac, GraphPad Software, www.graphpad.com. For analysis of Rab inhibition, reactions carried out with phosphatase alone were considered full activity; reactions with no PPM1H were considered zero activity. Thus, the amount of signal remaining was taken as the reciprocal of PPM1H activity.

## Data and materials availability

All reagents used in this study are available from commercial sources or repositories without restriction and Research Resource Identifiers for all reagents are provided in the key resource Table 2. All primary data have been deposited in Zenodo and can be found at https://doi.org/10.5281/zenodo.15427864. No code was generated for this study; all data cleaning, preprocessing, analysis, and visualization were performed using Adobe Photoshop 2025, Adobe Illustrator 2025, UCSF ChimeraX, GraphPad Prism (version 9), AlphaFold 3, and ImageJ2.

**Table 2:** Key resources

Key resources table					
Resource type	Resource name	Source	Identifier	New/reuse	Additional information
Antibody	Anti-Rab12 (mouse monoclonal)	Santa Cruz Biotechnology	sc-515613; Research Resource Identifier (RRID): AB_3101762	Reuse	(1:500)
Antibody	Anti-Rab10 (mouse monoclonal)	Abcam	ab104859; RRID: AB_10711207	Reuse	(1:1000)
Antibody	Anti-Rab10 (phospho T73) (rabbit monoclonal)	Abcam	Ab230261; RRID: AB_2811274	Reuse	(1:1000)
Antibody	Anti-PPM1H (rabbit monoclonal)	Abcam	Ab303536; RRID: AB_2941812	Reuse	(1:1000)
Antibody	IRDye 800CW Donkey anti-rabbit IgG	LI-COR	(926-32213; RRID: AB_621848)	Reuse	(1:10,000)
Antibody	IRDye 680RD Donkey anti-rabbit IgG	LI-COR	926-68073; RRID: AB_10954442	Reuse	(1:10,000)
Antibody	IRDye 800CW Donkey anti-mouse IgG	LI-COR	926-32212; RRID: AB_621847	Reuse	(1:10,000)
Antibody	IRDye 680RD Donkey anti-mouse IgG	LI-COR	926-68072; RRID: AB_10953628	Reuse	(1:10,000)
Bacterial and virus strain	*Escherichia coli* DH5α	Thermo Fisher	18258012	Reuse	
Bacterial and virus strain	*Escherichia coli* Rosetta DE3 pLys	Millipore	70956	Reuse	
Chemical, peptide, or recombinant protein	(18:0–20:4)PC (1-stearoyl-2-arachidonoyl-*sn*-glycero-3-phosphocholine)	Avanti Polar Lipids	850469	Reuse	
Chemical, peptide, or recombinant protein	(18:0–20:4)PI (1-stearoyl-2-arachidonoyl-*sn*-glycero-3-phosphoinositol)	Avanti Polar Lipids	850144	Reuse	
Chemical, peptide, or recombinant protein	(18:0–18:2)PS (1-stearoyl-2-linoleoyl-*sn*-glycero-3-phospho-L-serine)	Avanti Polar Lipids	840063	Reuse	
Chemical, peptide, or recombinant protein	(18:1)PI(4)P (1,2-dioleoyl-*sn*-glycero-3-phospho-(1′-myo-inositol-4′-phosphate)}	Avanti Polar Lipids	850151	Reuse	
Chemical, peptide, or recombinant protein	Cholesterol sulfate	Avanti Polar Lipids	700016	Reuse	
Chemical, peptide, or recombinant protein	(18:1) DGS NTA (Ni^2^+)	Avanti Polar Lipids	790404C	Reuse	
Chemical, peptide, or recombinant protein	Adenosine 5′-[γ-thio]triphosphate tetralithium salt	Sigma	A1388	New	
Chemical, peptide, or recombinant protein	Instant Blue Coomassie	Abcam	ab119211	Reuse	
Chemical, peptide, or recombinant protein	Bio-Rad Protein Assay Dye Reagent concentrate	Bio-Rad	5000006	Reuse	
Chemical, peptide, or recombinant protein	Zeba Spin Desalting Columns	Thermo Fisher Scientific	89882	Reuse	
Recombinant DNA	pET15b His SUMO Rab10 (Q68L) full length	Addgene	236720; RRID: Addgene_236720	Reuse	Human
Recombinant DNA	pET15b His-Mst3	MRC PPU Reagents and Services, U. Dundee	DU62980	Reuse	Human
Recombinant DNA	pET15b His SUMO Rab12 (Q101L) full length	Addgene	208371; RRID: Addgene_208371	Reuse	Human
Recombinant DNA	pET14b His Rab8A (Q67L) full length	Addgene	186014; RRID: Addgene_1860014	Reuse	
Recombinant DNA	pET15b His SUMO mNeon PPM1H	Addgene in progress	240002; RRID: Addgene_240002	New	Human
Recombinant DNA	pET15b His SUMO mNeon L66R PPM1H	Addgene in progress	240064; RRID: Addgene_240064	New	Human
Recombinant DNA	pET15b His SUMO mNeon R338A PPM1H	Addgene in progress	245124 RRID: Addgene_245124	New	Human
Recombinant DNA	pET15b 6His Rab8A	MRC PPU Reagents and Services U.Dundee	DU47349	Reuse	Human
Recombinant DNA	pET15b His SUMO mNeon Δ37 PPM1H	Addgene in progress	240065; RRID: Addgene_240065	New	Human
Protocol	Dephosphorylation of phosphorylated Rab GTPases by PPM phosphatases	protocols.io	dx.doi.org/10.17504/protocols.io.5jyl8d4j7g2w/v1	Reuse	
Protocol	Expression and purification of PPM1H phosphatase	protocols.io	dx.doi.org/10.17504/protocols.io.bu7wnzpe	Reuse	
Protocol	PPM1H purification from *E. coli*	protocols.io	dx.doi.org/10.17504/protocols.io.81wgbnp9ygpk/v1	Reuse	
Protocol	Preparation of unilamellar liposomes	protocols.io	dx.doi.org/10.17504/protocols.io.5qpvo3ke7v4o/v1	Reuse	
Protocol	Gibson Assembly Cloning	protocols.io	dx.doi.org/10.17504/protocols.io.eq2lyjwyqlx9/v1	Reuse	
Protocol	Sucrose density gradient coflotation of PPM1H and Rab10	protocols.io	dx.doi.org/10.17504/protocols.io.q26g79o2kvwz/v1	Reuse	
Software/code	UCSF ChimeraX	PMID: 1526424	RRID: SCR_015872	Reuse	
Software/code	GraphPad Prism	Prism 10, version 10.2.3	RRID: SCR_002798	Reuse	www.graphpad.com
Software/code	AlphaFold Server	AlphaFold 3	RRID: SCR_025885	Reuse	
Software/code	Image J2	https://imagej.net/software/imagej2/	RRID: SCR_003070	Reuse	
Software/code	MO affinity Analysis Software	NanoTemper	https://shop-us.nanotempertech.com/	Reuse	
Dataset	Allosteric regulation of the Golgi-localized PPM1H phosphatase by Rab GTPases modulates LRRK2 substrate dephosphorylation in Parkinson’s disease	Zenodo	10.5281/zenodo.15427864	New	Raw tabular data, original tiff files for blots, raw tabular data for all microscale thermophoresis binding experiments

## Supporting information

This article contains supporting information (11, 13).

## Conflict of interest

The authors declare that they have no conflicts of interest with the contents of this article.

## Figures and Tables

**FigS1:**
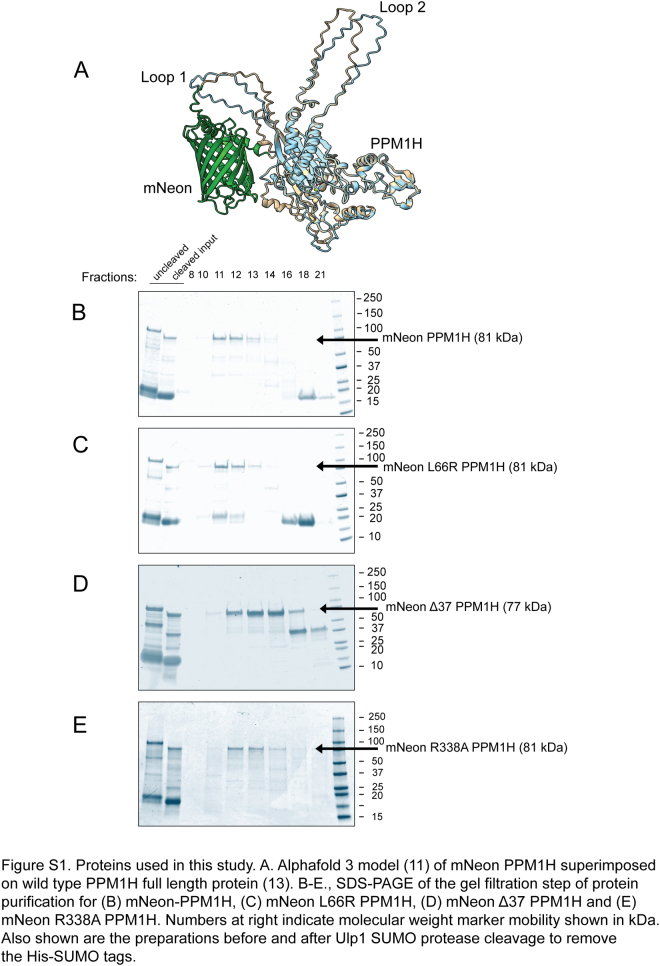


**FigS2:**
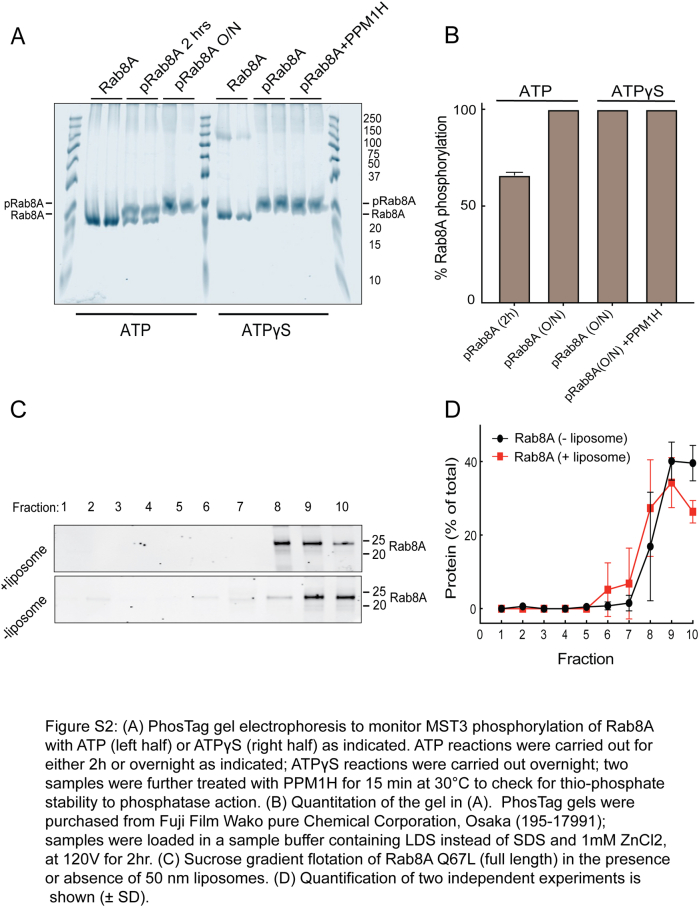


**FigS3:**